# The Combination of *Bacillus natto* JLCC513 and Ginseng Soluble Dietary Fiber Attenuates Ulcerative Colitis by Modulating the LPS/TLR4/NF-κB Pathway and Gut Microbiota

**DOI:** 10.4014/jmb.2402.02027

**Published:** 2024-05-10

**Authors:** Mingyue Ma, Yueqiao Li, Yuguang He, Da Li, Honghong Niu, Mubai Sun, Xinyu Miao, Ying Su, Hua Zhang, Mei Hua, Jinghui Wang

**Affiliations:** 1 Agronomy of Food Science and Technology, Yanbian University, Yanji 133002, Jilin, P.R. China; 2 Institute of Agro-product Process, Jilin Academy of Agricultural Science (Northeast Agricultural Research Center of China), Changchun 130033, Jilin, P.R. China

**Keywords:** *Bacillus natto*, ginseng dietary fiber, ulcerative colitis, LPS/TLR4/NF-κB, gut microbiota

## Abstract

Ulcerative colitis (UC) is an inflammatory bowel disease (IBD) that is currently difficult to treat effectively. Both *Bacillus natto* (BN) and ginseng-soluble dietary fiber (GSDF) are anti-inflammatory and helps sustain the intestinal barrier. In this study, the protective effects and mechanism of the combination of *B. natto* JLCC513 and ginseng-soluble dietary fiber (BG) in DSS-induced UC mice were investigated. Intervention with BG worked better than taking BN or GSDF separately, as evidenced by improved disease activity index, colon length, and colon injury and significantly reduced the levels of oxidative and inflammatory factors (LPS, ILs, and TNF-α) in UC mice. Further mechanistic study revealed that BG protected the intestinal barrier integrity by maintaining the tight junction proteins (Occludin and Claudin1) and inhibited the LPS/TLR4/NF-κB pathway in UC mice. In addition, BG increased the abundance of beneficial bacteria such as *Bacteroides* and *Turicibacter* and reduced the abundance of harmful bacteria such as *Allobaculum* in the gut microbiota of UC mice. BG also significantly upregulated genes related to linoleic acid metabolism in the gut microbiota. These BG-induced changes in the gut microbiota of mice with UC were significantly correlated with changes in pathological indices. In conclusion, this study demonstrated that BG exerts protective effect against UC by regulating the LPS/TLR4/NF-κB pathway and the structure and metabolic function of gut microbiota. Thus, BG can be potentially used in intestinal health foods to treat UC.

## Introduction

Ulcerative colitis (UC) is a prevalent type of inflammatory bowel disease (IBD). It damages the colonic lining and can lead to weight loss, blood in the stool, and even colon cancer [1]. Currently, aminosalicylic acids dominate the list of UC treatments. Traditional treatments cause fever as a side effect, and functional foods, such as probiotics and dietary fiber, can reduce intestinal inflammation and have been utilized as alternatives.

Synbiotics are combinations of probiotics and prebiotics. They can simultaneously actualize the physiological activity of probiotics and the effects of prebiotics to encourage growth [2]. Synbiotics reduce colon inflammation by lowering reactive oxygen species and increasing antioxidant enzyme levels. Several animal studies have shown that probiotics and prebiotics can reduce inflammatory response [3] and modulates the intestinal microflora [4]. Although some studies have confirmed that synbiotics can positively impact human health, the protective role of different probiotics combined with specific prebiotics (synbiotics) in inflammatory bowel disease remains unclear, indicating a wide range of research perspectives for synbiotics in the treatment of UC.

*Bacillus subtilis* can reduce lipopolysaccharide (LPS)-induced intestinal damage by promoting gut microbiota growth [5]. It can also enhance intestinal barrier integrity and decrease colonic inflammation by attenuating nuclear factor (NF-κB) activation [6]. Dietary fiber has been demonstrated to regulate gut microbiota. We previously showed that *Bacillus natto* JLCC513 is a potential probiotic strain that regulates gut microbiota disturbances and reduces intestinal barrier dysfunction [7]. Our team also found that ginseng-soluble dietary fiber (GSDF) has a prebiotic effect, which could regulate the gut microbiota and encourage the growth of probiotics [8]. These findings motivated us to investigate whether the combination of *B. natto* JLCC513 and ginseng-soluble dietary fiber (BG) could reduce inflammation in UC mice.

In this study, we investigated the effects of a novel synbiotic combination BG made of *B. natto* JLCC513 and ginseng-soluble dietary fiber on DSS-induced UC mice for the first time and analyzed its mechanism of action on the LPS/TLR4/NF-κB pathway and its impact on gut microbiota. Our study offers a research idea for examining the mechanism of action of synbiotics in alleviating UC and lays the groundwork for creating a new synbiotic diet for the prevention of colitis.

## Materials and Methods

### Animals and Materials

SPF grade seven-week-old male C57BL/6 mice (20~25 g) were purchased from Liaoning Changsheng Biotech Co. (China). Animal Production License number: No. SCXK (Liao) 2020-0001.

DSS (CAS# 9011-18-1) was provided by Shanghai YuanYe Biotech Co. (China). *Bacillus natto* JLCC513 (CGMCC No: 20625) was identified and preserved by the food microbiology laboratory of the Jilin Academy of Agricultural Sciences and preserved in the China General Microbiological Culture Collection Center (China). Ginseng soluble dietary fiber (GSDF) was extracted and obtained in the food microbiology laboratory of Jilin Academy of Agricultural Sciences (China), according to the method of Hua *et al*. (2020). Superoxide dismutase (SOD) and malondialdehyde (MDA) assay kits were purchased from Nanjing JianCheng Bioengineering Institute (China). The ELISA kits of IL-6, IL-10, IL-1, TNF-α, and LPS were all purchased from RuiDaHengHui Technology Development Co. (China). Other non-separately labeled reagents were of analytical grade.

### Bacterial Strains and Culture Conditions

Before the experiment, the glycerol tube conserved bacterial solution stored in a -80°C refrigerator (Haier Co., China) inoculated with 2% inoculum and cultured in LB culture medium at 37°C and 175 rpm for 12 h as the first activation (Vertical Constant-Temperature Shaker, Jiangsu Xinchunlan Scientific Instrument Co.). The second activation was inoculated 3% inoculum in tapered vials containing LB culture medium and incubating at 37°C for 6 h. The number of viable bacteria was determined by standard plate counting method and used when it reached 1×10^10^ CFU/ml. The cultured bacterial solution was poured into a 50 ml centrifuge tube and centrifuged (4,000 ×*g*) at 4°C for 10 min. The supernatant from the centrifuge tube was poured to leave the bacterial body in the centrifuge tube. Bacterial body were then washed three times using sterile phosphate buffer (PBS, pH = 7.4) to remove the medium liquid residue, centrifuged (4,000 ×*g*) at 4°C for 10 min again. The bacterial cells were re-suspended in sterile normal saline for gavage.

### Extraction of Ginseng Soluble Dietary Fiber

Extraction of ginseng soluble dietary fiber was performed following the Hua *et al*. (Hua *et al*. 2020). Briefly, the ginseng residue was extracted by enzymatic hydrolysis methods. The solid residue was washed twice with distilled water and 95% of ethanol. The supernatant, collected with the residue cleaning water, was filtered through 0.45 μM millipore filter and precipitated out by injecting four volumes of ethanol at 4°C for 24 h. The precipitate was filtered again, dissolved in distilled water, freeze drying and then crush, is the ginseng soluble dietary fiber (GSDF).

### Grouping of Animals and Administration of Drugs

Animal experiments were approved by the Laboratory Animal Management and Ethics Committee of the Institute of Agro-product Process, Jilin Academy of Agricultural Sciences (NO. JNK2023-010-01). C57BL/6 male mice were acclimatized at a constant temperature of 20–26°C, relative humidity of 40%–60%, and a 12 h cycle of light and dark for one week. The animals were randomly divided into six groups: normal control group (ND), colitis model group (DSS), *B. natto* JLCC513 group (BN), Ginseng soluble dietary fiber group (GSDF), *B. natto* and GSDF low-dose group (BGL, Same doses as the BN and GSDF), and *B. natto* and GSDF high-dose group (BGH), with 6 animals in each group. The experiment was divided into two phases: the first intervention period and the modeling period. During the first 14 days of the intervention period, the ND and DSS groups were given free access to sterile water, and all groups were given free access to ordinary maintenance feed. The BN group were given 200 μl of *B. natto* bacterial solution with a concentration of 1 × 10^10^ CFU/ml. The GSDF group were given 1.25% ginseng soluble dietary fiber drinking water. The BGL and BGH groups were given low-dose (1.25%, w/v) and high-dose (5%, w/v) drinking water of GSDF and 200 μl of BN bacterial solution with a concentration of 1×10^10^ CFU/ml by gavage.

After the 14-day intervention period and the 9-day modeling period, all mice, except for the ND group mice whose water and feeding intake unchanged, were given 5% (w/v) DSS aqueous solution freely every day, and maintenance chow was ingested freely. The feces were collected aseptically one day before the end of the intervention period and one day before the end of the modeling period, and stored at -80°C refrigerator.

At the end of the modeling period, the mice were fasted for 12 h. Blood was taken from the eyeball, and cervical dislocation. The colon tissue was quickly dissected and the length was measured. Some of the colon tissues were fixed with 4% paraformaldehyde fixative solution (Coolaber, SL1830, China), and the residual tissues were stored at -80°C refrigerator.

### Assessment of Disease Activity Index (DAI)

During the experiment, the weight and feces of the mice were observed every day. A test for hidden blood in the stool was carried out using a qualitative kit for detecting fecal occult blood (Leagene Biotechnology, TC0511, China).

The DAI scoring criteria according to Li *et al*. (Table 1) [9]. 

### Serum and Colon Tissue Antioxidant and Inflammatory Cytokines Analysis

The levels of SOD, MDA, IL-1, IL-6, IL-10, TNF-α, and LPS in serum were detected according to the assay kit’s instructions.

Colon tissue was homogenized with 9 times normal saline for 10 min. The levels of IL-1, IL-6, IL-10, and TNF-α in the supernatant were detected according to the ELISA kit instructions.

### Histological Analyses

The colon tissue was fixed with 4% paraformaldehyde fixative solution for 48 h, rinsed with running water, dehydrated with gradient ethanol, transparent with xylene, permeabilized with wax, embedded, deparaffinized and sectioned, stained with hematoxylin-eosin (HE) and alcian blue, respectively, and the histological structure of the colon was observed microscopically and photographed, and tissue damage scoring for colon tissue sections of each group, according to Dieleman *et al*. (Table 2) [10].

### Western Blot

The method mentioned above was used to extract the colon protein from the buffer solution of RIPA and to measure the protein quantification with the BCA Protein Kit (Beyotime, P0010, China). Electrophoretic isolate protein and transferred to membranes for 2 h and sealed for 1.5 h in 5% skim milk. The membranes were then incubated with the primary antibodies occludin (Wanleibio, WL01996, China), claudin-1 (Proteintech, 13050-1-AP, China), TLR4 (Proteintech, 66350-1-lg, China), NF-κB p65 (Proteintech, 10745-1-AP, China), Phospho-IκB Alpha (Proteintech, 2349-1-RR, China), IκB Alpha (Proteintech, 10268-1-AP, China) or β-actin (Beyotime, AF5003, China) at 4°C overnight. Wash with TBST, then incubate with the HPR (Beyotime, A0208, China) at room temperature for 1 h. Finally, using a SuperECL Plus Kit (UElandy, S6009, China) to exhibit the band. Bands were quantified using ImageJ software.

### 16S rRNA Gene Sequencing and Data Analysis

We performed the sequencing on the Illumina-NovaSeq-6000 platform at Personal Biotechnology Co. (China). The 0.2 g mouse fecal sample was taken to extract the total DNA of the intestinal microbiota using the QIAamp DNA Investigator Kit (Qiagen, Germany) and in strict accordance with the instructions, and the DNA concentration and purity were determined by using a NanoDrop Ultra-Micro Spectrophotometer. A PCR instrument was utilized to amplify the highly variable region V3-V4 series of the 16S rRNA gene of the sample bacteria, and the bacterial universal primers were used with forward primer 341F (5'-CCTAYGGGRBGCASCAG- 3') and reverse primer 806R (5'-GGACTCNNGGGTATCTAAT-3'). The purified amplification products were used to construct libraries on the Illumina-NovaSeq-6000 platform (Illumina, USA) according to standard operating procedures. The fastq files obtained from sequencing were quality-filtered using QIIME2 (2019.4). Fulllength module to remove repetitive sequences; use cluster_size module to cluster high-quality sequences OTUs (Operational Taxonomic Units) at 97% similarity level and output representative sequences and OTU table respectively. Finally, singletons OTUs (*i.e.*, OTUs with an abundance of 1 in all samples) were removed from the OTU table. The Greengenes database (Release 13.8, http://greengenes.secondgenome.com/) was selected for species taxonomic annotation of bacterial 16S rRNA genes. Single-sample diversity (α-diversity) and betweengroup diversity (β-diversity) analyses were performed based on the relative abundance of OTUs. Venn diagrams used to visualize the intersection of datasets between different groups were drawn via an online plotting website (https://en.wikipedia.org/wiki/Venn_diagram). Clustering results were calculated for each sample as well as for each taxonomic unit using R scripts to present a heat map of species composition for each group in the form of an interaction plot. A table of absolute abundance of taxonomic units at the phylum, order, order, family, and genus at the taxonomic level generated from the unsampled flat ASV/OTU table was used to call the "classify_samples_ncv" function in q2-sample-classifier to perform a Random Forest Analysis to infer the representative species strains of each group.

### Statistical Analysis

All experiments were repeated three times in parallel, and the data were processed by Microsoft Excel 2007 software. The results were expressed as mean ± standard deviation. SPSS 22.0 software was used for mathematical statistical analysis, and one-way ANOVA was used for intergroup comparisons, with significant differences at *p* < 0.05 and highly significant differences at *p* < 0.01. Statistical results were plotted by GraphPad Prism 8.0 software.

## Results

### Effect of BG on Colonic Inflammation in UC Mice

**Fig. 1:**
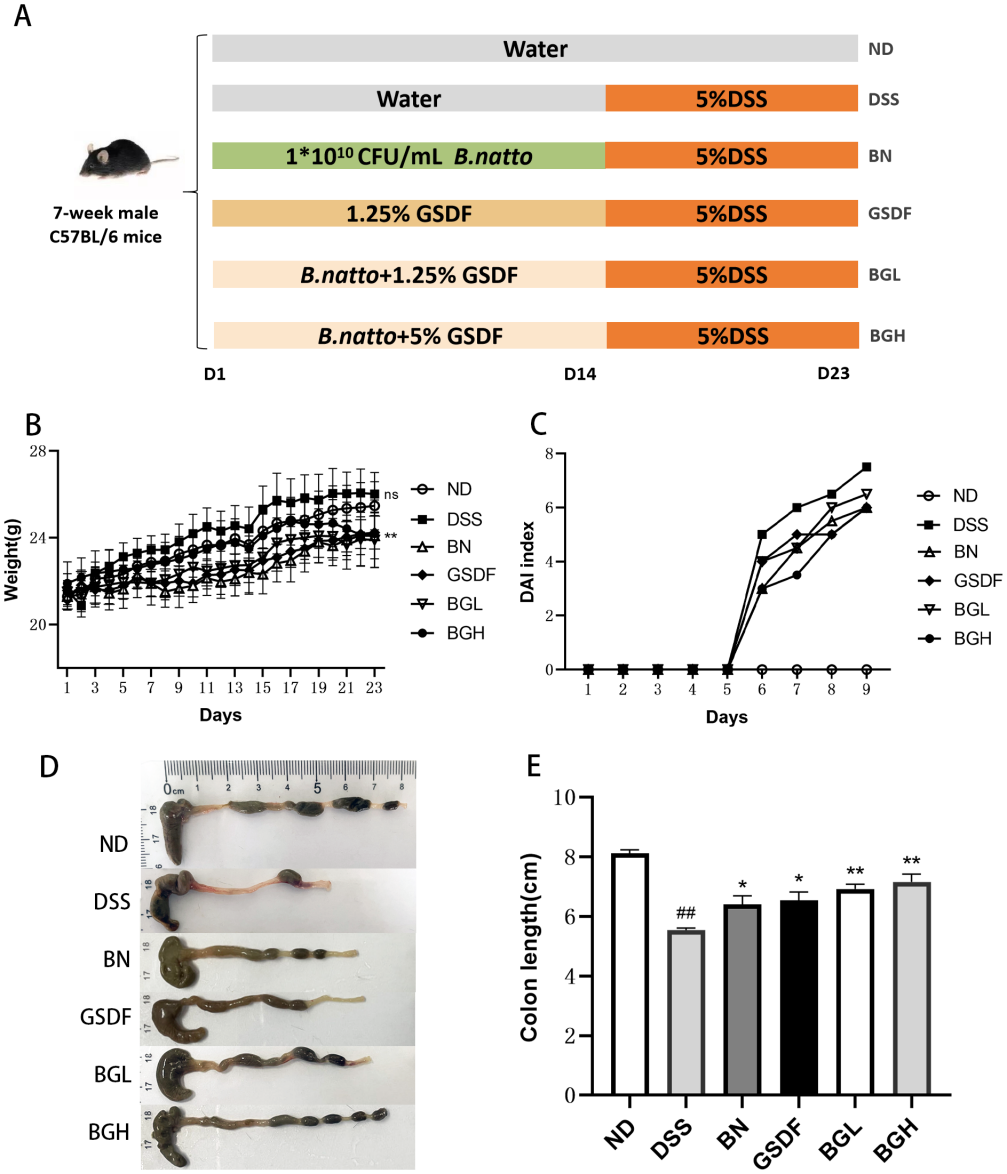
Symptoms of DSS-induced colitis in mice were alleviated by BG. (**A**) Experimental design protocol. (**B**) Body weight of mice during colitis. (**C**) Disease activity index (**DAI**) during colitis in mice. (**D, E**) Typical images of mouse colon and statistical analysis of colon length in each group. (Compared with the DSS group, *p < 0.05, **p < 0.01. Compared with ND group, ##*p* < 0.01)

According to the experimental design protocol (Fig. 1A), a gavage of *B. natto* (BN), ginseng-soluble dietary fiber (GSDF) drinking water, or a combination of BN and GSDF was administered during the pre-drying period, followed by 5% DSS drinking water for modeling. Although the body weight tended to increase in all groups throughout the administration period, there were signs of leveling off in the DSS group in the later stages of modeling (Fig. 1B). The DAI scores were significantly higher in the DSS group than in the ND group. However, the DAI scores decreased after the intervention in the BN, GSDF, and two BG groups; this decrease was more pronounced in the BGH dose group (Fig. 1C). In addition, the BN, GSDF, and BG groups showed significantly increased colon lengths after the intervention compared with that in the DSS group. The BG group showed a significant difference (Fig. 1D, E; *p* < 0.01). These results indicated that BG was more effective than BN or GSDF alone in preventing clinical symptoms in UC mice.

### Effect of BG on Colon Histopathology in UC Mice

**Fig. 2:**
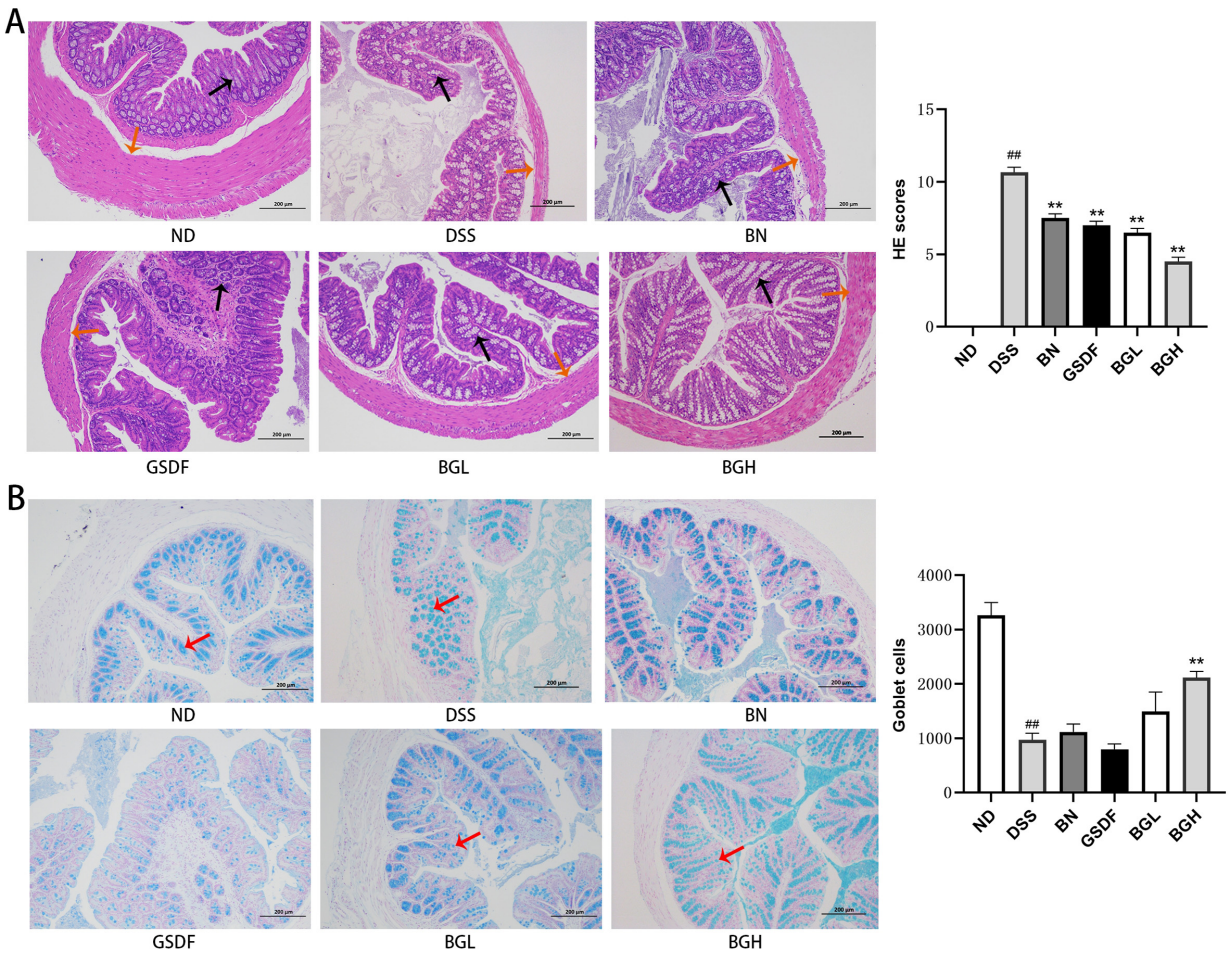
Histopathologic sections of the colon of the UC mice. (**A**) HE staining and HE scores. (**B**) Alisin blue staining and goblet cells. (Black arrow is crypts, orange arrow is muscular layer, red arrow is goblet cells)

In the ND group, the muscular layer of the colon, epithelial cells, and goblet cells were intact; no inflammatory cells were infiltrated; the glands were regular; and villi were well-arranged and well-defined. The DSS group had a marked inflammatory reaction in the colon compared to that of the ND group; the inflammation in the DSS group was marked by a large area of mucosal ulceration, decreased muscular layer thickness, and increased HE scores. After intervention with BN, GSDF, and BG, the colon HE scores decreased significantly. In addition, epithelial cell integrity and tissue damage improved significantly after the intervention in the BG group (Fig. 2A).

Alcian blue staining showed crypt loss, with a significant decrease in the large number of goblet cells in the colons of UC mice. However, BGH effectively alleviated this structural damage (Fig. 2B). These results suggest that BG can prevent tissue damage in UC mice more effectively than BN or GSDF separately.

### Effect of BG on Antioxidant and Anti-Inflammatory Capacity of UC Mice

**Fig. 3:**
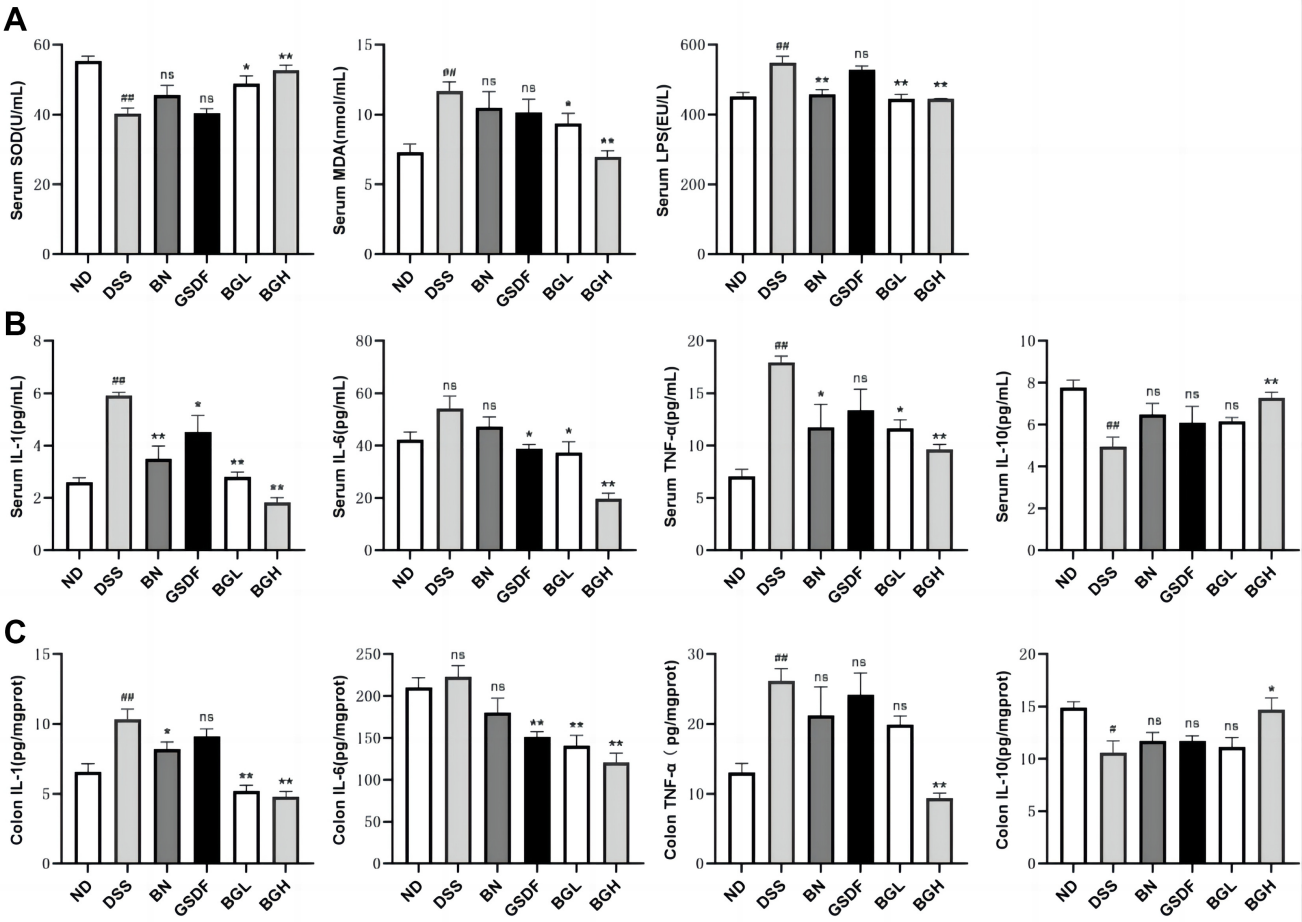
Effects of BG on oxidizing factors and inflammatory factors in UC mice. (**A**) Serum LPS levels, SOD activity, and MDA levels. (**B**) Serum IL-1, IL-6, IL-10 and TNF-α levels. (**C**) Colon IL-1, IL-6, IL-10 and TNF-α levels. (Compared with the DSS group, *p < 0.05, **p < 0.01. Compared with the ND group, #*p* < 0.05, ##*p* < 0.01.)

The anti-inflammatory and antioxidant effects of BG in UC mice were analyzed using oxidative and inflammatory factors. Compared with those in the ND group, serum LPS levels were abnormally elevated (*p* < 0.01), SOD activity was considerably decreased (*p* < 0.05), and MDA levels were elevated (*p* < 0.01) in the DSS group; these abnormalities recovered to near-normal levels after BG intervention, which was more effective than BN or GSDF (Fig. 3A; *p* < 0.05). Compared with those in the ND group, the DSS group showed a considerable increase in serum and colon IL-1, IL-6, and TNF-α levels and a decrease in IL-10 levels. This trend was significantly and more effectively reversed in the BG group than in the BN or GSDF groups in a dose-dependent manner compared with their changes in the DSS group (Fig. 3B and C; *p* < 0.05).

### Effect of BG on the Intestinal Barrier in UC Mice

**Fig. 4:**
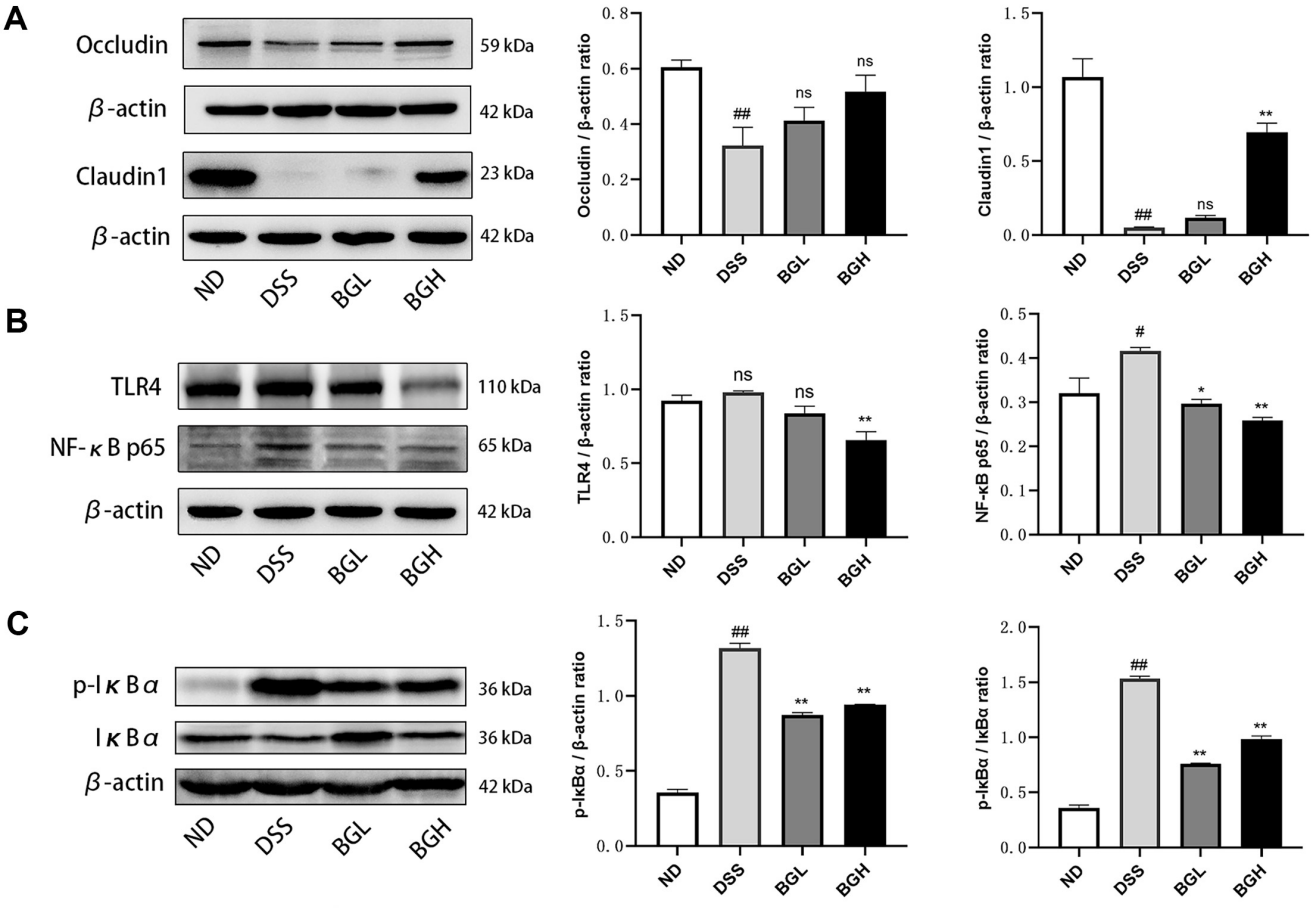
Effect of BG on colonic protein expression in UC mice. (**A**) Representative bands of colonic barrier proteins Occludin and Claudin1. (**B**) Representative bands of colonic inflammatory proteins TLR4 and NF-κB p65. (**C**) Representative bands of colonic proteins p-IκBα and IκBα. (Compared with the DSS group, *p < 0.05, **p < 0.01. Compared with the ND group, #*p* < 0.05, ##*p* < 0.01)

Damage to the intestinal barrier is associated with the development of colitis [11]. According to the above research, BG has more obvious advantages than BN or GSDF. Therefore, we focused on the mechanism of action of BG in the intestinal barrier and intestinal inflammatory signaling pathways. Compared with those in the ND group, the protein expression of Occludin and Claudin1 was reduced in the DSS group. A significantly higher expression of these proteins was observed in the BG group (Fig. 4A). In conclusion, BG was effective at protecting colonic epithelial cells and increasing the expression of tight junction proteins, with this improvement being more pronounced at higher doses.

The LPS-elevating activated TLR4 to trigger downstream signaling pathways, leading to the phosphorylation and subsequent degradation of IκBα, and the releasement of NF-κB. Compared with ND Group, the protein expression of p-IκBα and NF-κB p65 were up-regulated and the ratio of p-IκBα/IκBα was significantly increased in the DSS group (*p* < 0.05). After the intervention of BG, the abnormal expression of these proteins were significantly inhibited (Fig. 4B and C; *p* < 0.01) .

### Effect of BG on the Diversity and Structure of Gut Microbiota in UC Mice

**Fig. 5:**
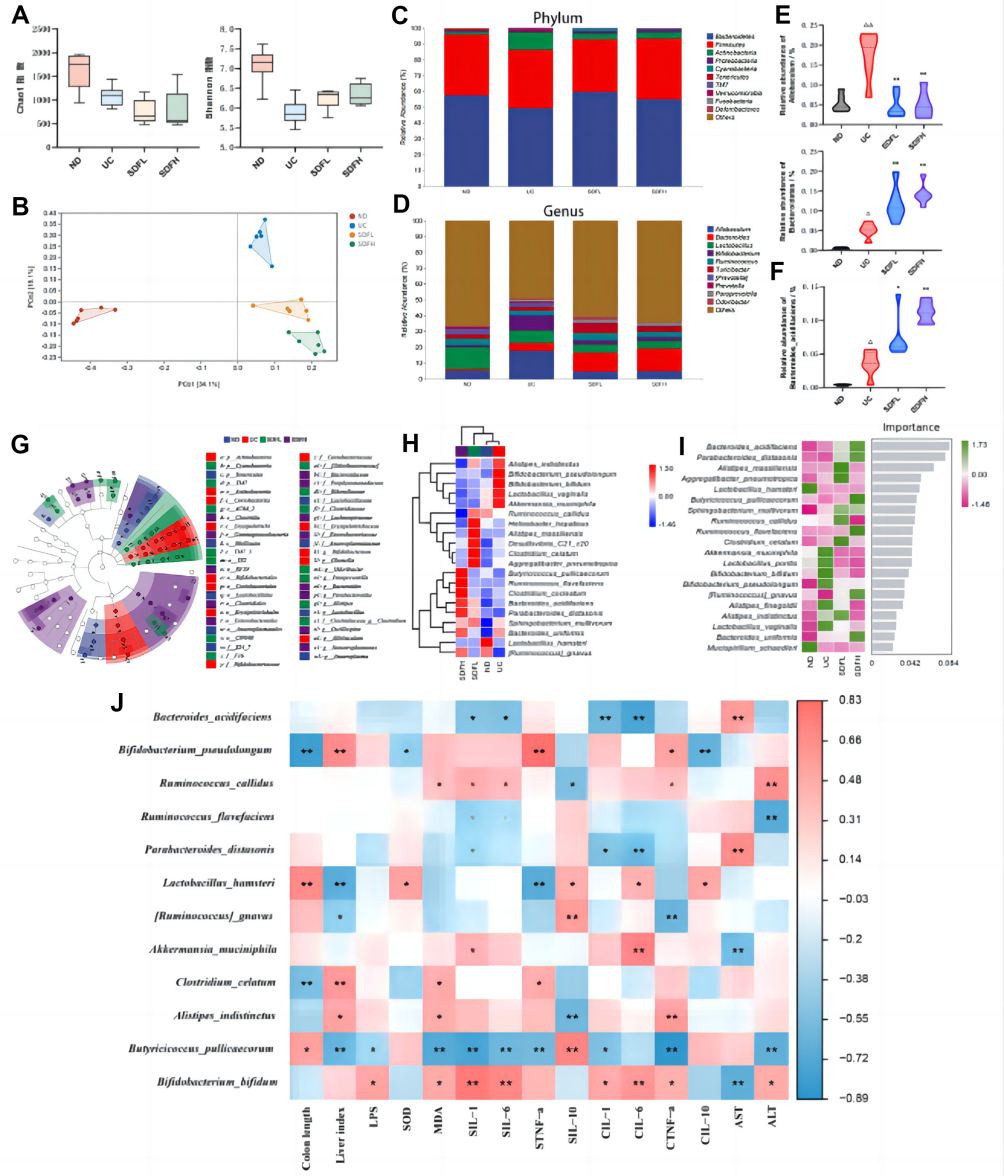
Effects of BG on the diversity, species abundance, and composition of characteristic strains of intestinal flora in UC mice. (**A**) Alpha diversity analysis (Chao1 and Shannon index). (**B**) Beta diversity principal coordinates analysis (PCoA) based on Bray-Crutis distance. (**C**) Relative abundance of microbiota at the phylum level. (**D**) Relative abundance of microbiota at the genus level. Relative abundance of *Allobaculum*, *Bacteroides* (**E**) *Bacteroides* acidifaciens (**F**) (**G**) Marker species taxonomic LefSe (LDA Effect Size) analysis. Node size corresponds to the mean relative abundance of the OTU; hollow nodes represent OTUs with non-significant differences between groups, while solid nodes represent OTUs with higher abundance and significant differences between groups. letters identify the names of taxonomic units with significant differences between groups. (**H**) Heat map of species composition at the species level. (**I**) Random forest model analysis of marker species. Importance on the right side indicates that from top to bottom species are of decreasing importance to the model, and it can be assumed that these species at the top of the importance scale are marker species for differences between groups. (**J**) Spearman's correlation analysis between intestinal flora and indicators of enterocolitis. (UC means DSS, BN/SDFL means BGL, BN/SDFH means BGH. Compared with the UC group, *p < 0.05, **p < 0.01. Compared with the ND group, Δ*p* < 0.05, ΔΔ*p* < 0.01)

We evaluated the effects of BG on the diversity and structure of gut microbiota in UC mice. Alpha diversity analysis showed that the balance of the gut microbiota was disrupted, and the richness and diversity of the gut microbiota were altered in the UC group. BG altered this trend to some extent (Fig. 5A; SDFL indicates BGL, SDFH indicates BGH). PCoA of beta diversity showed (Fig. 5B) significant inter-community differences in the UC group compared to that in the ND group and indicated that BG intervention altered the structure of the intestinal microbial community. Taxonomic composition analysis at the phylum (Fig. 5C) and genus (Fig. 5D) levels revealed a decrease in the abundance of *Bacteroidota* and an increase in the abundance of *Firmicutes* in the UC group; BG intervention altered the abundance of the above groups. Further, genus-level analysis revealed that BG decreased the abundance of *Allobaculum*, increased the abundance of *Ruminococcus*, *Turicibacter* (Fig. 5D), and *Bacteroides* (Fig. 5E) significantly. At the species level, the SDFH group significantly increased the relative abundance of *Butyricicoccus pullicaecorum* (Fig. 5F). In addition, the marker species of the UC group were concentrated with *Bifidobacteriales* of *Actinobacteria*, whereas the marker species of the SDFL group shifted to *Cyanobacteria* and were dominated by *Alistipes*. The marker species of the SDFH group deviated from *Cyanobacteria* toward *Clostridia* and were dominated by *Parabacteroides* (Fig. 5G). Species-level composition heat maps showed that the UC group had a higher abundance of *Bifidobacterium pseudopodium*, *Bifidobacterium bifidum*, and *Lactobacillus vaginalis* than the other groups. *Alistipes massiliensis*, *Clostridium celatum*, *Aggregatibacter pneumotropica*, and *Ruminococcus callidus* were the most abundant in the SDFL group. *Ruminococcus flavefaciens*, *Clostridium cocleatum*, *Butyricicoccus pullicaecorum*, *Parabacteroides distasonis*, and *Bacteroides acidifaciens* were abundant in the SDFH group (Fig. 5H). The signatures of intergroup differences in high abundance were further analyzed using a random forest model (Fig. 5I). *Akkermansia muciniphila* and *Lactobacillus pontis* were the top two signatures in the UC group and best represented the structural characteristics of the microbiota of this group. *Alistipes massiliensis* and *Aggregatibacter neurotropic* were the top two signatures in the SDFL group and best represented its microbiota structural features. *Bacteroides acidifies* and *Parabacteroides distasonis* were the top two signatures in the SDFH group and best represented the microbiota structural features of this group. We further explored the relationship between the intestinal microbiota and indicators of enteritis using Spearman’s correlation analysis (Fig. 5J). The signature strain *Butyricicoccus pullicaecorum* of the SDFH group showed a significant positive correlation with the colon length and the IL-10 level and a significant negative correlation with the liver index, liver function, LPS, MDA, IL-1, IL-6, and TNF-α levels. These results suggest that BG can regulate colon length, liver function, and LPS levels and reduce oxidative factors and inflammation levels by altering the diversity and structure of the gut microbiota.

### Effect of BG on the Metabolic Function of Gut Microbiota in UC Mice

**Fig. 6:**
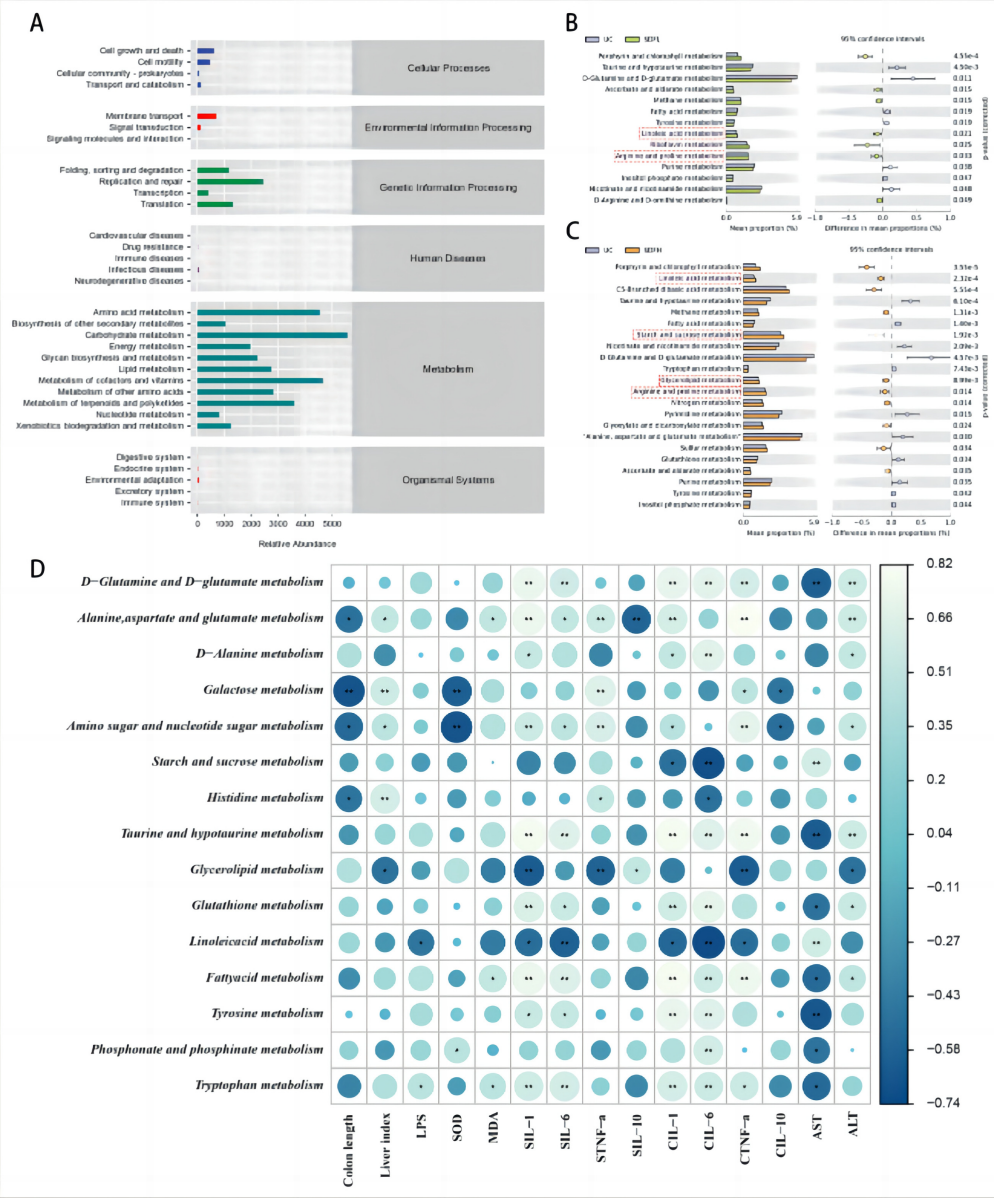
Effects of the BG on the metabolic function of intestinal flora in UC mice. (**A**) Relative abundance of KEGG signaling pathway functional gene expression in each group. (**B**) Metabolic pathways showed significant differences in functional gene expression abundance in the UC group vs. the SDFL group. (**C**) Metabolic pathways showed significant differences in functional gene expression abundance in the UC group vs. the SDFH group. (**D**) Spearman correlation analysis between metabolic pathways and indicators of enteritis. (UC means DSS, SDFL means BGL, SDFH means BGH)

DSS-induced functional changes in the gut microbiota were inferred using a PICSTR-based approach. The overall levels of gene expression in each group in the primary signaling pathway were similar (Fig. 6A). Among the secondary signaling pathways, the metabolism-related pathways showed significant changes. Further analysis of the differential pathways in the gut microbiota showed that, compared to those in the UC group, the SDFL group showed increased abundance of genes related to linoleic acid metabolism, and arginine and proline metabolism. The abundance of genes related to linoleic acid metabolism, starch and sucrose metabolism, glycerolipid metabolism, and arginine and proline metabolism in the gut microbiota increased significantly in the SDFH group (Fig. 6B and 6C). In summary, metabolic pathways may result in changes in the gut microbiota of UC mice. We further explored the relationship between the metabolic pathways and enteritis indicators using Spearman’s correlation analysis (Fig. 6D). Linoleic acid metabolism was positively correlated with colon length and IL-10 level and negatively correlated with LPS, MDA, IL-1, IL-6, and TNF-α levels. This suggests that BG--regulated linoleic acid metabolism may be involved in regulating colon length, lipopolysaccharide, and oxidative and inflammatory factor levels, ultimately slowing the pathogenesis of ulcerative colitis in mice.

## Discussion

UC is a persistent inflammatory disease. The composition and structure of the gut microbiota in patients with UC is damaged and that disequilibrium in the gut microbiota is the primary cause of UC. Probiotics, prebiotics, antibiotics, fecal microbiota transplantation, and a nutritious diet may help maintain a balanced environment for the gut microbiota. When combined with prebiotics (usually substances that are not digested or absorbed by the intestine), probiotic therapy has the potential to ameliorate illness. This probiotic-prebiotic composition, which can promote the growth of beneficial bacteria in the intestine, is often referred to as a synbiotic. Further, the variety of prebiotic ingredients in synbiotics can promote the growth of gut microbial metabolites. Therefore, the development of new synbiotic combinations is a valuable direction for gut health research.

In 2005, researchers first used a combination of *Bifidobacterium longum* (a probiotic isolated from healthy rectal epithelium) and an inulin-fructooligosaccharide substrate to treat patients with UC for one month and discovered that the clinical manifestations of all patients improved [12]. In recent years, researchers have used synbiotics with six distinct probiotics and fructooligosaccharides for the treatment of patients with mild UC for eight weeks, and these treatments have significantly improved clinical activity in patients [13]. Our research provides new possibilities for synbiotic combinations that can effectively treat UC.

*B. natto* is a subspecies of *Bacillus subtilis* and is one of the 40 probiotic strains approved by the U.S. Food and Drug Administration (FDA) [14]. *B. subtilis* can suppress the growth of UC and related colon cancer [15], control the host immune response [16], and maintain a balance of beneficial microbiota in the intestine [17]. However, few studies have investigated the UC-fighting mechanism of certain *B. natto* strains. According to previous studies, *B. natto* alters the structural composition of gut microbiota in mice and ameliorates metabolic disorders by controlling the expression of genes related to para and Srebp-1c, thereby preventing obesity [18]. This suggests that *B. natto* possesses anti-colitis properties. Ginseng was added to the list of dietary supplements in the Dietary Supplement Health and Education Act of 1994 by the FDA. It is a popular dietary supplement and functional food and has a positive immunomodulatory effect [19]. Our team's previous research demonstrated that GSDF is an acidic heteropolysaccharide (4.42% glucuronic acid), and its primary monosaccharide constituents include glucose (58.03%), galactose (14.50%), and mannose (2.84%) [8]. Therefore, the UC-treating effectiveness of BG in this investigation is based on the combined action of *B. natto* JLCC513 and GSDF. Different doses of glucose influence intestinal inflammation to varying degrees and a moderate dose of glucose (6% w/v) can alleviate colitis by inducing TERG cells [20]. Simultaneously, it was also shown that mannose can prevent mitochondrial dysfunction and tight junction protein disruption. It can effectively protect the intestinal epithelial cells from DSS-induced damage and can be used synergistically with mesalazine to treat colitis [21]. In conclusion, GSDF, composed of glucose and mannose as the main monosaccharides, can combine with *B. natto* JLCC513 to protect the intestinal barrier and regulate the immune response. Shinde *et al*. observed similar results; the combination of *Bacillus coagulans* and sugarcane fiber significantly enhanced the expression of intestinal tight junction proteins, regulated serum levels of inflammatory factors, and produced high concentrations of SCFAs. Thus, the symptoms of colitis in the mice were alleviated [22].

We also discovered that BG inhibited the LPS/TLR4/NF-κB pathway by exerting significant antioxidant and anti-inflammatory activities. Lipopolysaccharide (LPS) binds to TLR4 and releases TNF- α, which causes inflammation through TNFR1 signaling. However, IL-10 produces anti-inflammatory effects against inflammatory factors, reducing the oxidative stress [23]. TLR4 is mainly expressed on the cell surface and in nuclear endosomes of immune cells. After the damage caused by DSS treatment, TLR4 activation on macrophages contributes to their shift toward an inflammatory phenotype and consequently their release of inflammatory factors, including TNF-α, IL-1β, and IL­6, [24]. When oxygen free radicals, antigens, IL-1β and TNF-α stimulate cells, IκBα, an inhibitor of NF-κB, is phosphorylated by IKK complex rapidly. Due to the spatial conformational changes, the p-IκBα is recognized and degraded rapidly by 26S proteasomes. Because of p-IκBα degradation, the nuclear localization sequence of NF-κB is exposed and is transferred from the cytoplasm to the nucleus, then activates transcription of related genes and enhances expression of inflammatory cytokines [25]. In our study, DSS changed the expression level of regulatory protein p-IκBα, and then the TLR4/NF-κB pathway is regulated and affected. After BG intervention, TNF-α, IL-1, IL-6 and this pathway were all inhibited. The results suggested that BG had anti-inflammatory and antioxidant effects, and that the LPS/TLR4/NF-κB pathway was important for the alleviation of colitis by BG.

Additionally, BG modulated the dysbiosis of the gut microbiota, which boosted the abundance of good bacteria, such as *Bacteroides* and *Turicibacter*, and reduced the abundance of harmful bacteria, such as *Allobaculum*. The *Firmicutes*/*Bacteroidetes* ratio was reduced after BG intervention (Fig. S2), which may be attributed to its anti-inflammatory effect. The conventional probiotic *Akkermansia muciniphila* was abnormally enriched in the model group, which led to an imbalance in the intestinal flora and worsened inflammation. In contrast, after SDF intervention, the levels of BG returned close to those of the normal group, indicating that BG has a beneficial impact in maintaining the intestinal ecosystem. Although *A. muciniphila* can over-consume mucus, leading to intestinal barrier damage and inflammation, an appropriate amount of *A. muciniphila* not only protects the mucus layer against pathogens but also affects the metabolic immunity of the gut [26, 27]. Growth of the *Allobaculum* could exacerbate colitis [28]. When *Allobaculum* enters the mucous layer, it induces an inflammatory response in intestinal epithelial cells (IECs) and releases serum amyloid A [29, 30]. Furthermore, *Allobaculum* may be an important driver of pathological intestinal inflammation in humans [28]. This study demonstrated that the abundance of *Allobaculum* in UC mice was dramatically reduced by BG treatment. This could be a potential therapeutic target of BG in the treatment of inflammatory diseases.

BG also increases specific gut microbiota. *Alistipes* are anaerobic bacteria that exist mainly in the intestines of healthy people [31]. As an anti-inflammatory bacterium, *Alistipes* can reduce intestinal permeability and colitis [32]. Additionally, *Alistipes* can expand glucose to produce acetate and propionate. However, this genus plays a dominant role in disease modulation or may simply have a bystander role or an inducible role with other gut microbes in response to this problem. It is necessary to verify this through additional experiments. *Clostridium* can ferment dietary fibers in the gut to produce SCFA, which are important sources of energy for IECs [33]. *Clostridium butyricum* produces butyrate, which protects the intestinal epithelium, promotes IL-10 secretion, and restores tight junction protein expression [34]. *Butyricoccus pullicaecorum* is an important source of butyrate. Butyrate has various beneficial properties contributing to the maintenance of intestinal health. It is a major source of energy for colon cells [35], reduces colonic oxidative stress [36], inhibits pro-inflammatory cytokine release [37], and favors intestinal barrier function [38]. Luo *et al*. also found that *B. pullicaecorum* is involved in restoring the Th17/Treg balance and improving the symptoms of ulcerative colitis in mice [39].

*Bacteroides acidifaciens* is one of the primary commensal bacteria in the large intestine and is the signature strain of the SDFH group that was enriched during the intervention period (Fig. S3). *B. acidifaciens* may contribute to lower levels of IL-6 and TNF-α [40], promotion of IgA production [41], improvement of intestinal immune function, protection against intestinal pathogens, and reduction of the incidence of inflammatory bowel disease [42]. In addition, it is a major species involved in the lipid metabolism pathways of the gut microbiota [43]. Crucial lipid metabolic pathways for preventing UC include the metabolism of linoleic acid and arachidonic acid. Arachidonic acid is mostly catalyzed to prostaglandin E2 (PEG2) by cyclooxygenase (COX); it can also be catalyzed to eicosapentaenoic acid by lipoxygenase (LOX), which has anti-inflammatory properties [44, 45]. Linoleic acid, a precursor of arachidonic acid, can also be metabolized by COX and LOX to produce HPODE and HODE, which have similar anti-inflammatory effects [46]. Notably, 10-hydroxy-cis-12-octalienoic acid (HYA), a metabolite of linoleic acid, regulates TNFR2 through the GPR40-MEK-ERK pathway and suppresses IEC injury and colitis in mice [47, 48]. In our study, correlation analysis showed that linoleic acid metabolism was positively correlated with colonic length and IL-10 level and negatively correlated with the LPS, MDA, IL-1, IL-6, and TNF-α levels. This implies that *B. acidifaciens* may be the key regulatory strain of BG in alleviating UC and that its essential metabolic pathway may involve linoleic acid metabolism. However, the physiological effects of linoleic acid are poorly understood. Further research is required to better understand the mechanism by which BG treats ulcerative colitis by regulating linoleic acid metabolism. These results suggest that the mechanism by which BG prevents and ameliorates UC involves the regulation of colon barrier function and expression of inflammatory signaling pathways, as well as changes in the composition and function of the gut microbiota.

## Conclusion

In this study, we investigated the protective effects and mechanisms of the combination of BN and GSDF (BG) in UC mice. The results showed that BG improved the disease activity index and colon length of UC mice more effectively than BN or GSDF. BG also protects the intestinal barrier integrity by maintaining the expression of tight junction proteins, which in turn improves intestinal permeability and reduces oxidative stress and abnormal inflammatory factor levels. In particular, BG effectively regulates inflammation-related signaling pathways LPS/TLR4/NF-κB. In addition, BG reversed the dysbiosis of the gut microbiota, effectively increasing the abundance of beneficial bacteria, such as *Bacteroides* and *Turicibacter*, and decreasing the abundance of harmful bacteria, such as *Allobaculum*. The metabolic pathways of the gut microbiota were altered, and linoleic acid metabolism may be a key pathway involved in the regulation of BG. More importantly, BG-induced changes in the gut microbiota of UC mice were significantly correlated with the changes in pathological indices. Therefore, our study provides preliminary evidence regarding the potential of the combination of *B. natto* JLCC513 and GSDF in the maintenance of intestinal health and provides a direction for the development of novel synbiotic foods for the prevention and mitigation of UC.

## Supplemental Materials

Supplementary data for this paper are available on-line only at http://jmb.or.kr.



## Figures and Tables

**Table 1:** DAI scores.

Score	Weight loss (%)	Stool consistency	Blood in stool
0	-	Normal	Normal (-)
1	1-5	Soft but shaped	Occult blood (+)
2	5-10	Soft and unable to form	Slightly Bloody stool (++)
3	10-20	Loose stools	Bloody stool (++++)

DAI score = (weight loss score + stool consistency score + blood in stool score)/3 (1)

**Table 2:** Standard for evaluation of histological injury.

Score	Inflammation	Lesion depth	Crypts destruction	Extent of disease%
0	-	-	-	-
1	Slightly	Mucosa layer	1/3	1-25
2	Medium	Submucosa	2/3	26-50
3	Seriously	Muscularis /serosa	100%	51-100
