# Supplementary material for: The Combination of Bacillus natto JLCC513 and Ginseng Soluble Dietary Fiber Attenuates Ulcerative Colitis by Modulating the LPS/TLR4/NF-κB Pathway and Gut Microbiota
Source: J Microbiol Biotechnol. 2024 May 10;34(6):1287–98. doi: 10.4014/jmb.2402.02027 (PMC11239422; doi:10.4014/jmb.2402.02027)
Supplement: Supplementary file 1 [file jmb-34-6-1287-supple.pdf]

J. Microbiol. Biotechnol. 2024. 34(6): 1287–1298

https://doi.org/10.4014/jmb.2402.02027

Supplementary Figures

The combination of *Bacillus natto* JLCC513 and ginseng soluble dietary fiber attenuates ulcerative colitis by modulating the LPS/TLR4/NF-κB pathway and gut microbiota

BG attenuates ulcerative colitis by modulating the LPS/TLR4/NF-κB pathway and gut microbiota

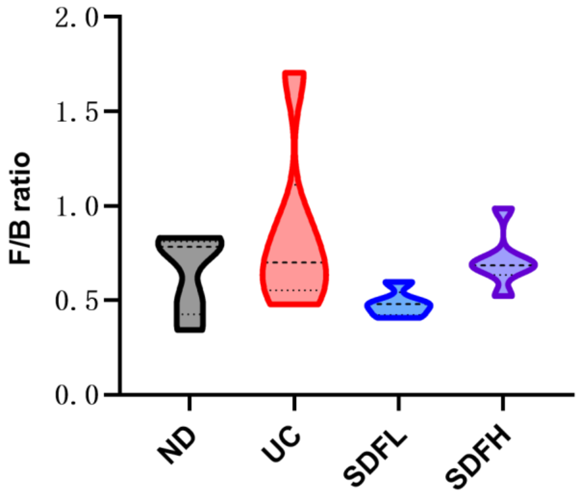

Fig. S1. Firmicutes/Bacteroidetes (F/B) ratio of gut microbiota in UC mice.

(UC means DSS, SDFL means BGL, SDFH means BGH)

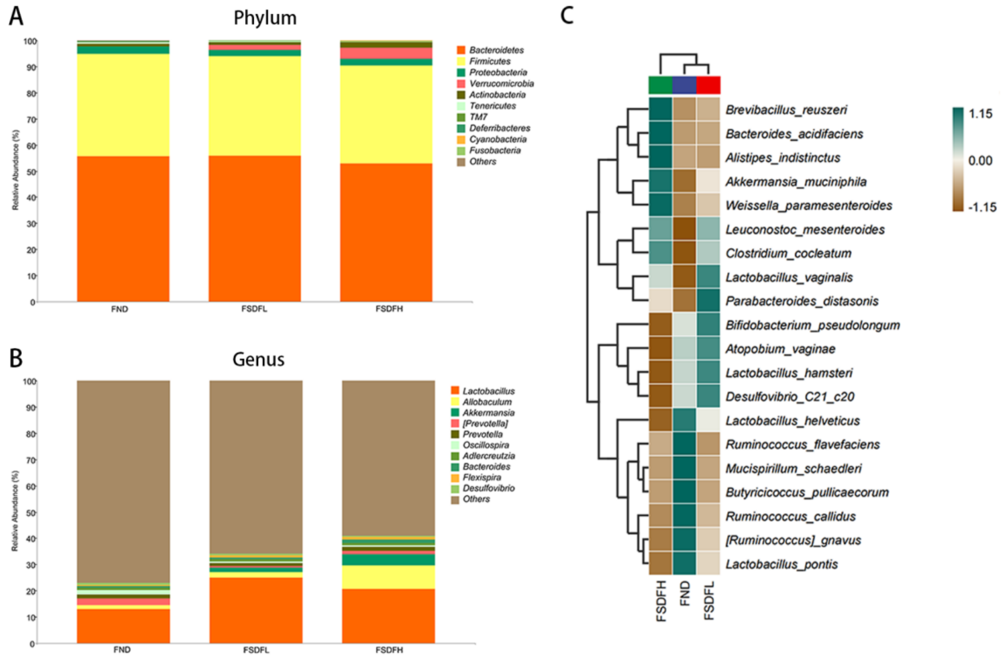

Fig. S2. Effects of BG on the strain abundance, and composition of characteristic strains of gut microbiota in heath mice. (A) Relative abundance of microbiota at the phylum level**. (B)** Relative abundance of microbiota at the genus level. **(C)** Heat map of composition at the species level. (ND group of intervention period (FND). BGL group of intervention period (FSDFL). BGH group of intervention period (FSDFH))
